# Identification of an endogenous retroviral envelope gene with fusogenic activity and placenta-specific expression in the rabbit: a new "syncytin" in a third order of mammals

**DOI:** 10.1186/1742-4690-6-107

**Published:** 2009-11-27

**Authors:** Odile Heidmann, Cécile Vernochet, Anne Dupressoir, Thierry Heidmann

**Affiliations:** 1 Unité des Rétrovirus Endogènes et Eléments Rétroïdes des Eucaryotes Supérieurs, CNRS UMR 8122, Institut Gustave Roussy, 39 rue Camille Desmoulins, F-94805 Villejuif, and Université Paris-Sud, Orsay, F-91405, France

## Abstract

**Background:**

*Syncytins *are envelope genes of retroviral origin that have been co-opted by the host to mediate a specialized function in placentation. Two of these genes have already been identified in primates, as well as two distinct, non orthologous genes in rodents.

**Results:**

Here we identified within the rabbit *Oryctolagus cuniculus*-which belongs to the lagomorpha order- an envelope (*env*) gene of retroviral origin with the characteristic features of a bona fide *syncytin*, that we named *syncytin-Ory1*. An *in silico *search for full-length *env *genes with an uninterrupted open reading frame within the rabbit genome first identified two candidate genes that were tested for their specific expression in the placenta by quantitative RT-PCR of RNA isolated from a large set of tissues. This resulted in the identification of an *env *gene with placenta-specific expression and belonging to a family of endogenous retroelements present at a limited copy number in the rabbit genome. Functional characterization of the identified placenta-expressed *env *gene after cloning in a CMV-driven expression vector and transient transfection experiments, demonstrated both fusogenic activity in an *ex vivo *cell-cell fusion assay and infectivity of pseudotypes. The receptor for the rabbit syncytin-Ory1 was found to be the same as that for human syncytin-1, *i.e. *the previously identified ASCT2 transporter. This was demonstrated by a co-culture fusion assay between hamster A23 cells transduced with an expression vector for ASCT2 and A23 cells transduced with *syncytin-Ory1*. Finally, *in situ *hybridization of rabbit placenta sections with a *syncytin-Ory1 *probe revealed specific expression at the level of the junctional zone between the placental lobe and the maternal decidua, where the invading syncytial fetal tissue contacts the maternal decidua to form the labyrinth, consistent with a role in the formation of the syncytiotrophoblast. The *syncytin-Ory1 *gene is found in Leporidae but not in Ochotonidae, and should therefore have entered the lagomorpha order 12-30 million years ago.

**Conclusion:**

The identification of a novel *syncytin *gene within a third order of mammals displaying syncytiotrophoblast formation during placentation strongly supports the notion that on several occasions retroviral infections have resulted in the independent capture of genes that have been positively selected for a convergent physiological role.

## Background

Previous studies have identified two pairs of envelope (*env*) genes of retroviral origin that have been independently captured by their host for a role in placentation. In simians, *syncytin-1 *[1-3] and *syncytin-2 *[4,5] entered the primate genome 25 and >40 million years (My) ago, respectively. They retained their coding capacity in all the subsequent branches; *syncytin-1 *and *syncytin-2 *display placenta-specific expression, are fusogenic in *ex vivo *cell-cell fusion assays, and one of them displays immunosuppressive activity [6]. A pair of *env *genes from endogenous retroviruses (ERVs) were then identified in the mouse, named *syncytin-A *and *-B*, which share closely related functional properties although they have a completely distinct origin, showing a divergent sequence and a different genomic location compared to primate syncytins [7]. As found for the latter, *syncytin-A *and *-B *have the status of bona fide genes. They have been conserved since their entry into the Muridae genome, approximately 20 million years (My) ago; they display placenta-specific expression, mediate cell-cell fusion in *ex vivo *assays [7], and one of them is immunosuppressive [6]. Recently we have further unambiguously demonstrated via the generation of *syncytin-A *knockout mice that these genes are indeed essential for placentation, with a lack of cell-cell fusion observed *in vivo *at the level of the placenta of the knockout embryos, resulting in impaired maternal-fetal exchanges and death of the embryos at mid-gestation [8]. Therefore, it appears that on some occasions in the course of mammalian evolution, *env *genes from endogenous retroviruses have been "co-opted" by their host to participate in the formation of the syncytiotrophoblast layer, at the maternal-fetal interface, by mediating the fusion of mononucleated cytotrophoblasts.

A further question that we wanted to answer was whether mammals belonging to orders other than rodents and primates but possessing a placenta with a related architecture, *i.e. *with a syncytiotrophoblast layer in direct contact with maternal blood at the maternal-fetal interface, have also "captured" retroviral *env *genes to generate, in a convergent manner, this specific placental structure. Among mammals whose placenta displays such a structural organization at the maternal-fetal interface, *i.e. *with a haemochorial placenta, the lagomorpha order was selected over the two other orders which also possess a haemochorial placenta -*i.e. *the Insectivora (hedgehog) and Chiroptera (bats) [9]; this was done because one of its representatives, the rabbit (*Oryctolagus cuniculus*), has an already sequenced genome, and can be reared and investigated easily at different stages of gestation, and has a placental physiology that has been appropriately described [9-11].

Here, by combining *in silico *search for *env *genes within the rabbit genome, RT-PCR assays for their *in vivo *transcriptional activity in a large panel of tissues including the placenta, cloning of the candidate genes, *ex vivo *assays for their fusogenicity and, ultimately, *in situ *hybridization of placenta sections, we identify a new fusogenic and placenta-specific endogenous *env *gene, displaying all the characteristic features of a bona fide *syncytin *gene, that we named *syncytin-Ory1*. Although we demonstrate that the syncytin-Ory1 protein shares the same ASCT2 receptor in common with human syncytin-1, it is divergent from all four syncytins previously identified in rodents and primates and must therefore have been captured independently from a distinct ancestral retrovirus. The occurrence in a third order of Mammals of a new *syncytin *gene that is specifically expressed at the maternal-fetal interface within the placental junctional zone provides strong support to the notion that ERVs have played a convergent role in the recurrent emergence of syncytiotrophoblast-containing haemochorial placentae in the course of evolution.

## Results and Discussion

### *In silico *search for retroviral *env *genes within the rabbit (*Oryctolagus cuniculus*) genome

**Figure 1:**
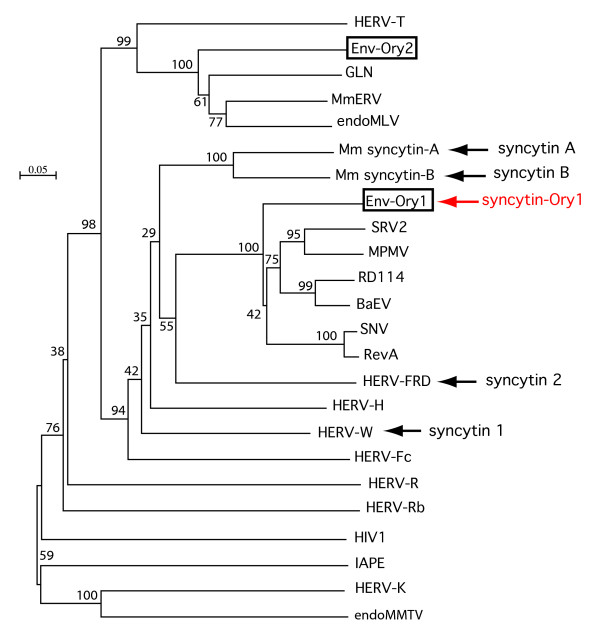
**Retroviral envelope protein-based phylogenic tree with positions of the identified rabbit Env-Ory1- and Env-Ory2, and of the human and mouse syncytins**. The tree was determined by the neighbor-joining method using envelope TM subunit sequences (see ref [12]) from murine and human ERVs, and infectious retroviruses. The horizontal branch length and the scale indicate the percentage of amino acid substitutions from the node. Percent bootstrap values obtained from 1,000 replicates are indicated.

To identify putative *env*-derived *syncytin *genes, we made use of the available rabbit genome sequence (low coverage 2× assembly of the *Oryctolagus cuniculus *genome, Ensembl May 2005 assembly, updated version 49) and of the method that we previously devised to screen the whole human and mouse genomes for such genes [7,12]. Basically, it makes use of the degenerate CKS17u consensus motif, associated with the immunosuppressive domain of retroviral envelope proteins, and is designed to match the majority of *env *genes of exogenous and endogenous origin [12]. Rabbit sequences from the Ensembl database were screened with this motif using the BIOMOTIF program, and only sequences with open reading frames (ORFs) longer than 1.5 kb were considered. Five ORF-containing sequences were obtained, four of which disclosed >98% nt identity. We named these four sequences Env-Ory1. The fifth sequence that was obtained was unrelated to Env-Ory1. We named this sequence Env-Ory2 (Figure 1). Analysis of the scaffold database identified the former ORF as belonging to a low-copy family of ERVs, most of which had an *env *gene that was interrupted by stop codons, deletions and/or truncations. Due to the low coverage of the available assembly, it could not be determined whether the identified ORF corresponds to distinct loci or to a single locus - in that case with distinct alleles.

### Transcription profile and identification of a placenta-specific envelope

**Figure 2:**
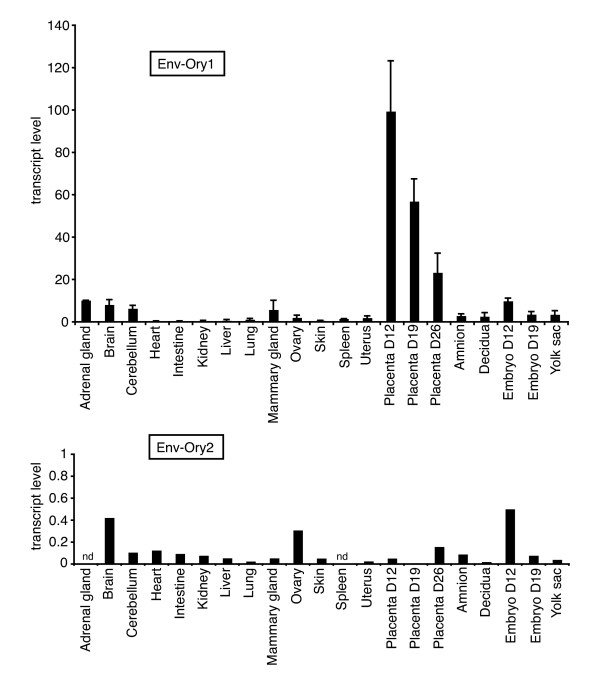
**Real-time quantitative RT-PCR analysis of Env-Ory1 and Env-Ory2 transcripts in rabbit tissues**. Transcript levels were normalized relative to the amount of 18S rRNA (arbitrary units). At least 3 samples per organ type were analyzed for Env-Ory1 (from different adult animals for non-fetal tissues; from a given litter for the embryos and placentae); one sample per organ type was analyzed for Env-Ory2.

Quantitative RT-PCR analysis of transcript levels for the two identified candidate *syncytin*s was performed using primers specific for each family of elements. As illustrated in Figure 2, the Env-Ory1-encoding gene has the characteristic profile of a bona fide *syncytin *gene, with high levels of expression in the placenta and very limited expression in other tissues. Expression in the placenta decreases with gestational age, with a four-fold reduction from day 12 to 26 (*i.e. *4 days before delivery). The other candidate gene encoding Env-Ory2 is expressed only limitedly (at least 100-fold lower), with no specific expression in the placenta, and was not considered further.

**Figure 3:**
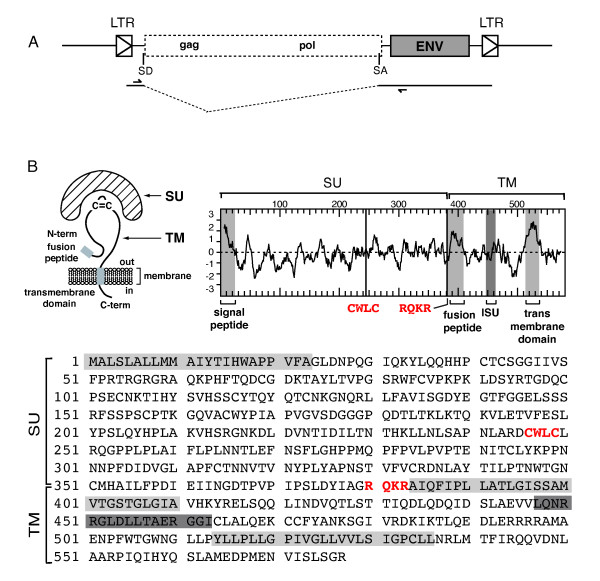
**Characterization of Env-Ory1**. (A) Schematic representation of the Env-Ory1-associated ERV with the LTRs and the splice sites for the sub-genomic *env *transcript indicated. (B) Schematic structure, hydrophobicity profile and primary sequence of the Env-Ory1 glycoprotein (deposited in GenBank [GenBank:GU196371]). The SU and TM subunits of the envelope protein are delineated, with a canonical furin cleavage site (RQKR; consensus: R/K-N-R/K-R) between the two subunits and the CWLC domain involved in SU-TM interaction indicated in red; the hydrophobic signal peptide and fusion peptide and the transmembrane domain are shaded in light gray, and the putative immunosuppressive domain (ISU) in dark gray.

Some of the sequence scaffolds reveal the Env-Ory1-encoding gene to be part of a proviral structure with degenerate but identifiable LTR, *gag *and *pol *gene sequences. A phylogenetic tree based on the envelope TM subunit (Figure 1) shows that this envelope protein is related to that of type-D retroviruses such as MPMV, BaEV and RD114, as similarly observed on the basis of a *pol*-based tree (not shown). A putative donor and acceptor splice site for the generation of a subgenomic *env *transcript can be identified according to http://www.cbs.dtu.dk/services/NetGene2, as classically observed for retroviruses. Their position and functionality were further ascertained by RT-PCR analysis of Env-Ory1-encoding transcripts in the placenta, using appropriate primers (see Methods and Figure 3).

Analysis of the amino-acid sequence of Env-Ory1 (consensus in Figure 3 of the four sequences in the database, also corresponding to that PCR-amplified, see below) displays the characteristic features of retroviral envelope proteins, with a putative signal peptide at the N-terminus, and a furin cleavage site (RQKR, consensus R/K-X-R/K-R) at amino acid 380 to generate the SU and TM subunits. The hydrophobicity plot of the TM subunit reveals a putative fusion peptide at the TM N-terminus, and a hydrophobic transmembrane domain.

### Assay for the fusogenic activity of Env-Ory1

**Figure 4:**
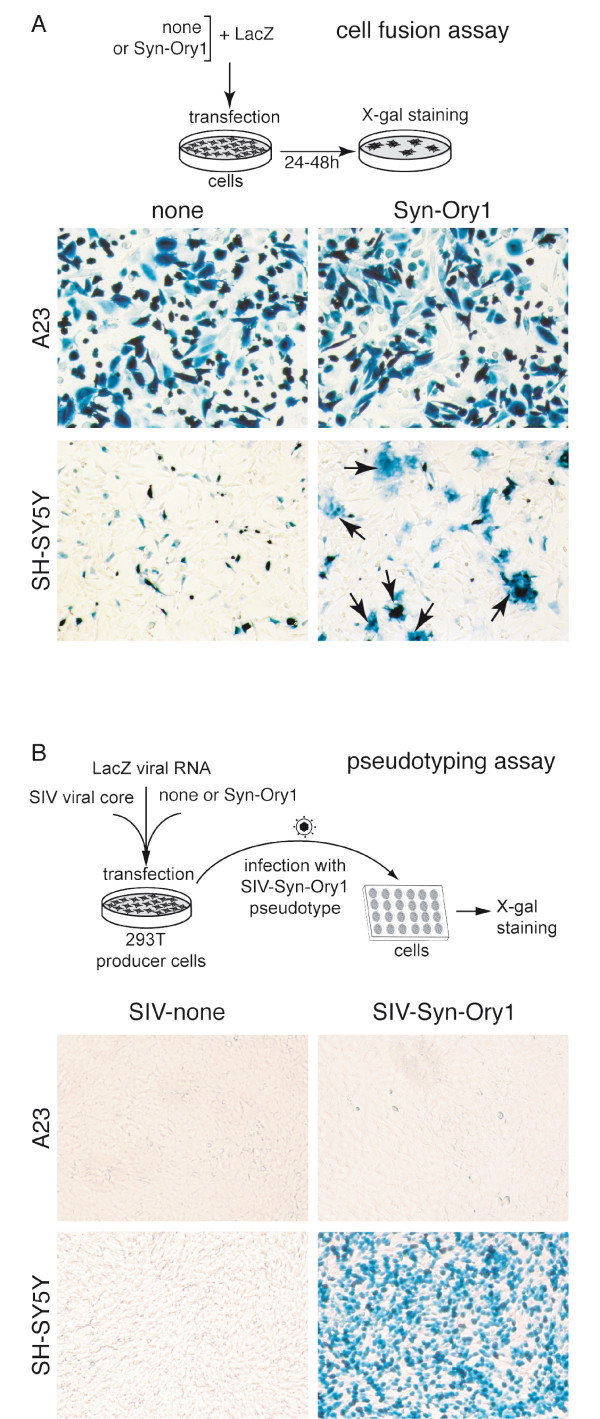
**Fusogenic activity of syncytin-Ory1**. (A) Assay for cell-cell fusion mediated by syncytin-Ory1. The indicated cell lines were transfected with an expression vector for syncytin-Ory1 or an empty vector (none) together with a LacZ expression vector. Cells were cultured for 1-2 days after transfection, fixed and X-gal-stained. Syncytia (arrows) were detected in *syncytin-Ory1*-transfected SH-SY5Y cells, with only mononucleated cells visible in the other cases. (B) Assay for cell infection mediated by syncytin-Ory1-pseudotyped virus particles. Pseudotypes were produced by cotransfection of human 293T cells with expression vectors for the SIV core, the syncytin-Ory1 protein (or an empty vector) and a *LacZ*-containing retroviral transcript. Supernatants were used to infect the indicated target cells, which were X-gal stained 3 days after infection. Abbreviation: Syn-Ory1, syncytin-Ory1.

Fusogenic activity of Env-Ory1 was assayed as previously described [4,5,7], by *ex vivo *assays in cells in culture for both detection of syncytia formation (cell-cell membrane fusion) and generation of infectious pseudotypes (virus-cell membrane fusion). The Env-Ory1-encoding sequence was first PCR-amplified from genomic DNA of *Oryctolagus cuniculus *and inserted into a CMV promoter-containing expression vector (see Methods). The plasmids were sequenced and those containing a full-length *env *gene ORF (that were actually >99.7% identical, and therefore most probably corresponding to a single sequence element) were assayed. As illustrated in Figure 4A, transient transfection of human SH-SY5Y neuroblastoma cells with the Env-Ory1-expressing vector triggers cell-cell fusion, as expected for a bona fide syncytin expressed in a cell line carrying its cognate receptor. Cell-cell fusion is not observed with the hamster A23 cells, which were therefore used as a control in the following assays for receptor identification.

**Figure 5:**
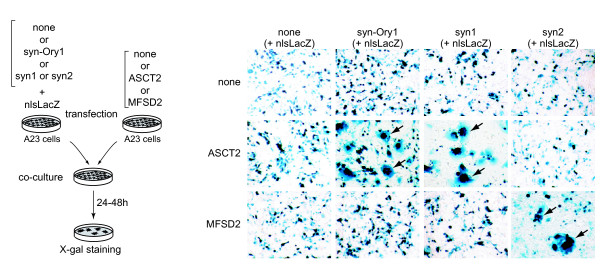
**Fusion assay between *ASCT2*-transduced and *syncytin-Ory1*-transduced co-cultured cells demonstrates that ASCT2 is the syncytin-Ory1 receptor**. Left panel: Cell-cell fusion was assayed upon independent transfections of a set of A23 cells with an empty vector (none) or an expression vector for either the syncytin-Ory1, syncytin-1 or syncytin-2 protein together with an nls-*LacZ *gene-expression vector, and another set of A23 cells with an expression vector for the syncytin-1 receptor ASCT2, the syncytin-2 receptor MFSD2 [13] or an empty vector (none). One day after transfection, cells were resuspended and pairs of transfected cells from each set were cocultured for 1-2 days, fixed and X-Gal stained. Right panel: Syncytia can be easily detected (arrows) for the syncytin-Ory1/ASCT2, syncytin-1/ASCT2 and syncytin-2/MFSD2 pairs, with only mononucleated cells visible in the other cases. Abbreviations: syn-Ory1, syncytin-Ory1; syn1, syncytin-1; syn2, syncytin-2.

Env-Ory1 can also form infectious pseudotypes, as expected from its retroviral origin. As illustrated in Figure 4B, pseudotypes generated with an SIV core are able to infect SH-SY5Y cells that are positive in the cell-cell fusion assay above, but not A23 cells. The profile of cells positive for infection was found to be similar to that observed for human syncytin-1 (data not shown), thus suggesting that Env-Ory1 could possibly use the same receptor (*i.e. *the neutral amino acid trasporter ASCT2, [2]). This point was investigated in the experiments illustrated in Figure 5 in which distinct pools of A23 cells transfected with either an ASCT2 or an Env-Ory1 expression vector (supplemented with a β-galactosidase expression vector) were mixed and assayed for cell-cell fusion. As shown in the figure, cell-cell fusion (as revealed by the presence of large LacZ+ syncytia) could be observed with the Env-Ory1 and ASCT2 pair (as well as with the ASCT2/syncytin-1 and MFSD2/syncytin-2 [13] pairs, used as positive controls) but not with any of the other combinations. This strongly suggests that Env-Ory1 uses the ASCT2 receptor, as does human syncytin-1. It is rather unexpected for two independently acquired - and distinct - retroviral envelopes to use the same cellular receptor. ASCT2 seems, however, to be a rather "successful" receptor being also the one used by a series of type-D infectious retroviruses, such as the primate MPMV, feline RD114, and avian SNV viruses [14,15] whose Env actually clusters with Env-Ory1 in phylogenic trees (Figure 1 for the TM domain and data not shown for SU). Finally, database screening indeed reveals the presence of an ASCT2 gene in the rabbit genome (mRNA accession number NM_001082378; 85% amino-acid identity with human ASCT2), and qRT-PCR demonstrates its expression in the rabbit placenta (data not shown). In conclusion, Env-Ory1 can be considered as a bona fide syncytin owing to its fusogenic activity and specific expression in the placenta, and be named Syncytin-Ory1.

### *In situ *hybridization of placenta sections

**Figure 6:**
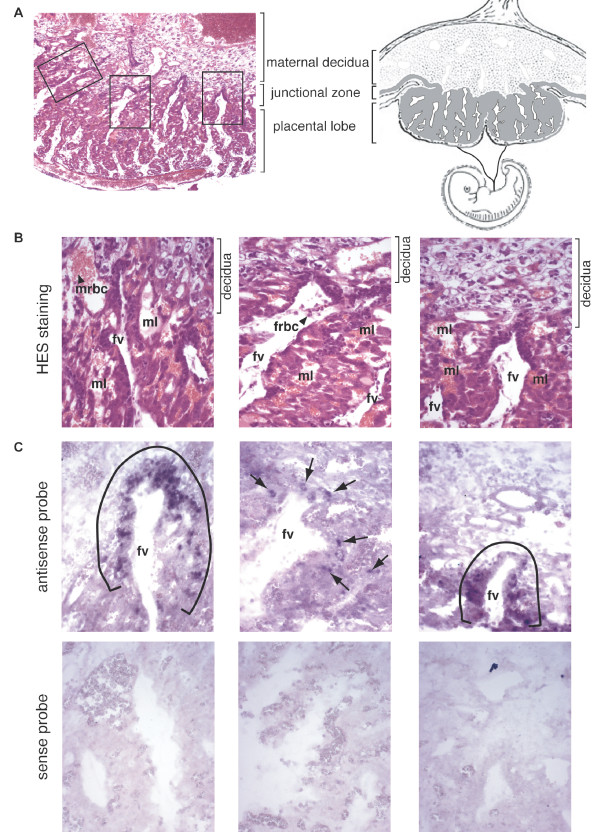
**Structure and in situ hybridization for *syncytin-Ory1 *expression of day 12 rabbit placenta: (A) Schematic representation of a rabbit placenta (right) and haematoxylin and eosin staining of a day 12 placenta section (left) with the 3 main layers of the placenta indicated**. (B) Higher magnification of the areas framed in A. Abbreviations: frbc: fetal red blood cell, fv: fetal blood vessel, ml: maternal blood lacuna, mrbc: maternal red blood cell. (C) *In situ *hybridization on sections of a day 12 rabbit placenta (serial sections of the HES in B) with digoxigenin-labeled syncytin-Ory1 sense (lower panel, negative control) and antisense (upper panel) riboprobes, revealed with an alkaline phosphatase-conjugated anti-digoxigenin antibody. Brackets and arrows highlight the positive labeling of trophoblast cells surrounding the invading fetal vessels in the junctional zone.

To further assess the physiological relevance of *syncytin-Ory1 *expression in the placenta, *in situ *hybridization experiments were performed on paraffin sections of placenta at day 12 of gestation, *i.e. *the stage showing maximum expression of *syncytin-Ory1 *by qRT-PCR (Figure 2). Figure 6A shows the representative architecture of rabbit placenta at day 12. Three main zones can be distinguished. The maternal decidua results from the modification of the uterus after implantation, and the placental lobe consists in a labyrinthine structure where fetal-maternal exchanges take place. In the labyrinth, fetal and maternal blood circulations are separated by 2 layers of trophoblasts (haemodichorial placenta). Between the decidua and placental lobe, a junctional zone can be observed where fetal vessels surrounded by invading fetal tissue contact the maternal decidua to form the labyrinth (Figure 6A, B). The invading fetal tissue has been described as a broad syncytial front toward the decidua, backed by and presumably formed from cellular cytotrophoblasts [9]. Thus, as soon as the fetal processes reach the maternal blood spaces, a haemodichorial structure is formed, with both a cellular and a syncytial trophophoblast layer separating maternal and fetal blood spaces (maternal lacuna, ml, and fetal vessels, fv). All of these characterize the definitive labyrinthine placenta [9,11].

A specific digoxigenin-marked antisense probe was synthesized for *syncytin-Ory1 *transcript detection, as well as the corresponding sense probe as a negative control. As shown in Figure 6C, specific labeling was observed only with the antisense probe (upper panels), but not with the control probe (lower panels). *Syncytin-Ory1 *expression is restricted to the junctional zone, where its expression (of variable intensity depending on the area, even within the same placental section) is limited to trophoblast cells surrounding the invading fetal vessels (brackets for heavily marked zones and arrows for punctuated domains in Figure 6C). Although we cannot formally discriminate between the syncytial trophoblast and the cellular cytotrophoblasts, the labeling profile is compatible with *syncytin-Ory1 *being expressed in the cytotrophoblast just before fusion takes place and/or in the newly formed syncytiotrophoblast (no labeling was detected in the placental lobe outside the junctional zone). These observations are consistent with a role for *syncytin-Ory1 *in the formation of the syncytiotrophoblast.

### *Syncytin-Ory1 *sequences are present in Leporidae but not in Ochotonidae

**Figure 7:**
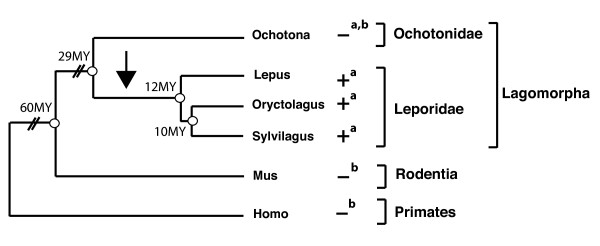
**Putative entry date of *syncytin-Ory1 *during lagomorph evolution**. Schematized phylogenetic tree with the evolutionary timeline of four lagomorph genus (adapted from [16]) and the rodent and primate outgroups depicted, with average divergence times indicated for the nodes. The presence of *syncytin-Ory1 *sequences in each genus, detected either by PCR experiments (a) or database screening (b), is indicated on the right.

Phylogenetic relationships in the order of Lagomorpha are illustrated in Figure 7 (adapted from [16]). The order includes 2 families, Ochotonidae (pikas) and Leporidae (hares and rabbits). We searched for *syncytin-Ory1 *genes in lagomorph species belonging to the Ochotonidae family (*Ochotona princeps*) and the Leporidae family (genus *Lepus*: *Lepus americanus*, *Lepus europaeus *and *Lepus starcki*; genus *Sylvilagus*: *Sylvilagus brasiliensis*, *Sylvilagus floridanus*). The domestic rabbit, *Oryctolagus cuniculus*, analyzed in this study, belongs to the genus *Oryctolagus *within the Leporidae.

Genomic DNA from these different species was PCR-amplified using a pair of primers for the *syncytin-Ory1 *ORF. A 1900 bp specific amplicon could be obtained from the 3 *Lepus *species, but neither from the *Sylvilagus *nor from the *Ochotona *genus members tested. However, a pair of primers internal to the ORF allowed the amplification of a 1430 bp PCR product from the 2 *Sylvilagus *species analyzed. Again, no specific amplification could be obtained from the *Ochotona princeps *genomic DNA. The PCR products obtained from *Lepus *and *Sylvilagus *were cloned and sequenced and showed >88% nucleotide identity with the *Oryctolagus cuniculus syncytin-Ory1 *gene sequence, and therefore most probably correspond to amplification of the same genes.

The absence of a *syncytin-Ory1 *gene in the Ochotona (as well as in the more ancestrally diverged rodent (mouse) and primate (human) orders) was confirmed by screening (using the BLAST program) the corresponding Ensembl databases.

Conclusively, the identification of *syncytin-Ory1 *genes in *Lepus *and *Sylvilagus *in addition to *Oryctolagus *indicates that gene capture most probably occurred prior to the divergence of the *Lepus *and *Oryctolagus/Sylvilagus *genera ~12 My ago [16]. The absence of a *syncytin-Ory1 *gene in *Ochotona princeps *suggests that this integration took place after the Leporidae and Ochotonidae divergence ~30 My ago [16].

## Conclusion

Here we have shown that within a placental mammal that has developed a haemochorial placenta with a syncytiotrophoblast layer at the maternal-fetal interface, such as the rabbit, a gene of retroviral origin can be identified which displays all the characteristic features of a bona fide *syncytin *gene. The identified *syncytin-Ory1 *has a cell-cell fusion activity and is expressed specifically in the placenta at a location consistent with a direct role in syncytiotrophoblast formation. *syncytin-Ory1 *can be found in a series of leporidae. Notably, the identified *env *gene is divergent from any other previously described genes. Thus, for a newly investigated mammalian order, we provide evidence that a retroviral *env *gene has been captured and has gained the status of a "*syncytin*" according to a process similar to that previously observed in the two other major orders where haemochorial placentae have emerged: the primates (*syncytin-1 *and *-2*) and the rodents (*syncytin-A *and *-B*).

It is therefore likely that such gene captures have arisen recurrently during the evolution of placental mammals and that *syncytin *genes will be found in other placental mammals displaying a syncytiotrophoblast organization. Ongoing investigations carried out on representative animals of the Carnivora order strongly support this hypothesis. An interesting question which remains to be answered is to determine which specific properties of the captured syncytins are responsible for the relative diversity observed in the physiology of mammalian placentation. Among species with hemochorial placentation in particular, most of the species (including human, rabbit and most rodents) have a single layer of syncytiotrophoblast, whereas a few others (Muridae) have a two-layered syncytiotrophoblast. Moreover, it will be of interest to determine whether placental mammals [such as Suidae (pig) or Equidae (horse)] which do not possess a syncytiotrophoblast layer at their maternal-fetal interface, are deprived of syncytins, or whether other functions of retroviral envelope proteins such as their immunosuppressive activity have driven the capture of a non-fusogenic *syncytin*-like gene. In this case, retroviral envelope proteins would be co-opted solely for an immunological role in relation with maternal-fetal tolerance. Experiments in progress with knock-in mice where *syncytin *genes have been mutated for immunosuppressive activity without impairment of fusogenicity may also help answer these questions.

## Methods

### Database screening and sequence analyses

Retroviral *env *gene sequences were searched by using the Biomotif program http://www.lpta.univ-montp2.fr/users/menes/bioMotif_html_doc/ref_Run.html and the degenerate universal CKS17u consensus motif [12] as a query. We made use of the available rabbit genome sequence (low coverage 2× assembly of the *Oryctolagus cuniculus *genome, Ensembl RABBIT May 2005 assembly, updated version 49). ORF-containing scaffolds (scaffold number and ORF-coordinates indicated) were: 131951(25124:26887), 15025(429:2192 reverse strand), 90528(17499:19256), 163359(7744:9486 reverse strand) for Env-Ory1, and 1093(1017:3035 reverse strand) for Env-Ory2. Multiple alignments were carried out by using the CLUSTALW program http://bioinfo.hku.hk/services/analyseq/cgi-bin/clustalw_in.pl. Phylogenic trees were constructed from alignments by using the neighbor-joining program within CLUSTALW and were viewed with the NJPLOT program.

The *Ochotona princeps *(Pika) genome low coverage 1.93× assembly (Ensembl OchPri2.0 June 2007, updated version 53), as well as of the human and mouse assemblies (from the Genome Reference Consortium) were also screened for the presence of the identified rabbit Env-Ory1 ORF sequence, using the BLAST programs at the National Center for Biotechnology Information http://www.ncbi.nlm.nih.gov/BLAST.

### Real-time RT-PCR

Env-Ory1 and Env-Ory2 mRNA expression was determined by real-time quantitative RT-PCR. Pregnant New Zealand white rabbit females obtained from INRA (Jouy-en-Josas, France) at various stages of gestation were sacrificed, and dissected organs were stored in liquid nitrogen. Total RNA was extracted from the frozen organs using the RNeasy RNA isolation kit (Qiagen). Reverse transcription was performed with 1 μg of DNase-treated RNA as in [17]. Real-time qPCR was with 5 μl of diluted (1:10) cDNA in a final volume of 25 μl by using SYBR Green PCR Master Mix (or Taqman Universal PCR Master Mix for 18S rRNA detection) (Applied Biosystems). PCR was carried out using an ABI PRISM 7000 sequence detection system. Primer sequences were as follows: 5'-GCTGTTTTTATGCTAACAAGTCC and 5'-GATAAAGGTCATCAGCCTATTGA for Env-Ory1 and 5'-CCTCTAAATGTCATCTTCACCAG and 5'-CTATTGGGACAGCAGTTCTAGTC for Env-Ory2,. The transcript levels were normalized relative to the amount of 18S rRNA (as determined with the primers and Taqman probe from Applied Biosystems). Samples were assayed in duplicate.

### *Syncytin-Ory1 *expression vector

The *syncytin-Ory1 *expression vector was constructed as follows: *syncytin-Ory1 *was PCR-amplified from genomic DNA of New Zealand white rabbit using the Accuprime DNA polymerase (Invitrogen) for 30 cycles. XhoI-containing primer sequences were: 5'-ATCACCTCGAGTGCTGGAATTGTTGTCATTGTTG and 5'-ATCACCTCGAGCGTCATTGGCTTACTGCTCATTT. After restriction with XhoI, the PCR product was cloned into the phCMV-G vector (GenBank accession AJ318514, gift F.-L. Cosset) opened with XhoI. Constructs were verified by sequencing.

### Cell fusion assay

Cell lines described in [13,18] were grown in DMEM medium supplemented with 10% fetal calf serum (Invitrogen).

For the self-fusion assay, cells seeded at 10^4 ^- 5 × 10^4 ^cells per well in 24-well plates were transfected by using the Lipofectamine kit (Invitrogen) with 0.2 μg of either the *syncytin-Ory1 *expressing or an empty vector, supplemented with 0.2 μg of a LacZ-expression vector (pCMV-β, Clontech). Syncytia were visualized by X-Gal staining 24 to 48 h after coculture.

For cell-cell fusion by the coculture assay, A23 cells were seeded at 5 × 10^5 ^cells per 60-mm dish. A set of dishes were transfected by using the Lipofectamine LTX kit (Invitrogen) with 5 μg of either an *ASCT2 *or an *MFSD2 *expression vector [13] or an empty vector, and another set were transfected with 2.5 μg of either a *syncytin-Ory1 *or a *syncytin-1 *or a *syncytin-2 *expression vector or an empty vector, each cotransfected with 2.5 μg of the nls-LacZ expression vector (R9SA, [19]). One day after transfection, 3.5 × 10^5 ^cells from each group of transfected cells were cocultured in 6-well plates. Syncytia were visualized by X-Gal staining 24 to 48 h after coculture.

### Pseudotyping assay

SIV virions pseudotyped with syncytin-Ory1 were produced by cotransfecting 8 × 10^5 ^293T cells with: 2.25 μg pSIV3+ (encoding SIV retroviral proteins except Env) [20]; 2.25 μg R9SA (a LacZ-marked defective SIV retroviral vector) [19]; and 0.5 μg of *syncytin-Ory1 *expression vector, using the Lipofectamine LTX transfection kit (Invitrogen). Supernatants from the transfected cells were harvested 48 h after transfection, filtered through 0.45 μm pore-size PVDF membranes, supplemented with Polybrene (4 μg/ml), transferred to target cells seeded in 24-well plates (5 × 10^4 ^- 8 × 10^4 ^cells per well) the day before infection, followed by spinoculation at 1200 × *g *for 2 h 30 min at room temperature. X-Gal staining was performed 3 days later.

### *In situ *hybridization

Freshly collected rabbit placentae (at day 12 of gestation) were fixed in 4% paraformaldehyde at 4°C, embedded in paraffin, and serial sections (4 μm) were either stained with haematoxylin and eosin or used for *in situ *hybridization. A PCR-amplified 1135 bp *syncytin-Ory1 *fragment (primers: 5'-AGACTGCGGAGATAAAACTGC and 5'-GTGGACCGCGATTCCTAGTC) was cloned into pGEM-T Easy (Promega) for *in vitro *synthesis of the antisense and sense riboprobes, generated with SP6 RNA polymerase and digoxygenin 11-UTP (Roche Applied Science). Sections were processed, hybridized at 42°C overnight with the riboprobes and incubated further at room temperature for 2 h with alkaline phosphatase-conjugated anti-digoxygenin antibody Fab fragments (Roche Applied Science). Staining was revealed with NBT and BCIP phosphatase alkaline substrates as indicated by the manufacturer (Roche Applied Science).

### Search for *syncytin-Ory1 *in other lagomorphs

Genomic DNAs from *Lepus americanus*, *Lepus europaeus*, *Ochotona princeps*, *Sylvilagus brasiliensis *and *Sylvilagus floridanus *were a gift from A. Surridge (Department of Zoology, University of Cambridge, UK). Genomic DNA from *Lepus starcki *was extracted from tissue given by F. Catzeflis (Laboratoire de Paléontologie, Université de Montpellier 2, France). Genomic DNAs were digested by Not I, which does not cut within the *syncytin-Ory1 *gene. PCRs were performed on 100 ng of DNA, using Accuprime Taq DNA Polymerase (Invitrogen) for 40 cycles (30 sec at 94°C, 30 sec at 50°C, 2 min at 68°C). The primers used were: 5'-TTCCTGAGGGCTCACTGATTAAC and 5'-GAAGGGGAGAGTCAGTTGTTGGAG (external to the ORF) or 5'-AGACTGCGGAGATAAAACTGC and 5'-gataaaggtcatcagcctattga (internal to the ORF). PCR products were then cloned in pGEM-T Easy vector (Promega) for subsequent sequencing. Primer sequences for splice site determination were: 5'-CTTGGGGTTCGAGCCTGT and 5'-TTGAGCACGGCCACGGCCAC, on each side of the putative splice donor (SD) and acceptor (SA) sequences, respectively (see Figure 3A).

## Competing interests

The authors declare that they have no competing interests.

## Authors' contributions

OH, CV, AD and TH designed research and drafted the manuscript. OH, CV and AD performed research. All authors read and approved the final manuscript.
